# Interfacial biomechanical properties of a dual acid-etched versus a chemically modified hydrophilic dual acid-etched implant surface: an experimental study in Beagles

**DOI:** 10.1186/s40729-018-0139-1

**Published:** 2018-09-27

**Authors:** Rainde Naiara Rezende de Jesus, Eunice Carrilho, Pedro V. Antunes, Amílcar Ramalho, Camilla Christian Gomes Moura, Andreas Stavropoulos, Darceny Zanetta-Barbosa

**Affiliations:** 1 Department of Periodontology, Faculty of Odontology, Malmö University, Carl Gustafs väg 34, 205-06 Malmö, Sweden; 2 IBILI, Faculty of Medicine, University of Coimbra, Av. Bissaya Barreto, Bloco de Celas, 3000-075 Coimbra, Portugal; 3 CEMUC, Mechanical Engineering Department, University of Coimbra, Pinhal de Marrocos, 3030-788 Coimbra, Portugal; 4 Department of Endodontics, Faculty of Odontology, Federal University of Uberlândia, Av Pará 1720, Bloco4LB, Campus Umuarama, Uberlândia, Minas Gerais 38405-900 Brazil; 5 Department of Oral and Maxillofacial Surgery and Implantology, Faculty of Odontology, Federal University of Uberlândia, Av Pará 1720, Bloco4LB, Campus Umuarama, Uberlândia, Minas Gerais 38405-900 Brazil

**Keywords:** Biomaterial, Dental implant, Removal torque, Removal energy, Connection stiffness, Implant roughness, Wettability, Dogs, New methodology assessment for implants

## Abstract

**Background:**

The high survival clinical success rates of osseointegration are requisites for establishing a long-term biomechanical fixation and load-bearing potential of endosseous oral implants. The objective of this preclinical animal study was to evaluate the effect of surface microtopography and chemistry on the early stages of biomechanical rigidity with a sandblasted, dual acid-etched surface, with or without an additional chemical modification (SAE-HD and SAE, respectively), in the tibia of Beagle dogs.

**Methods:**

Two pairs of implants, with the same macrogeometry but different surface technology ((a) dual acid-etched surface treatment with hydrochloric and sulfuric acid followed by microwave treatment and insertion in isotonic saline solution to increase hydrophilicity (SAE-HD) (test, *n* = 12) and (b) dual acid-etched surface (SAE) (control, *n* = 12)), were installed bilaterally in the proximal tibia of six Beagle dogs. In order to determine the effect of surface modification on biomechanical fixation, a test protocol was established to assess the torque and a complete set of intrinsic properties. Maximum removal torque (in N cm) was the primary outcome measure, while connection stiffness (N cm/rad) and removal energy (× 10^−2^J) were the secondary outcome measures and were assessed after 2 and 4 weeks in vivo. A general linear statistical model was used and performed for significant differences with the one-way ANOVA followed by Tukey post hoc test (*P* < 0.05).

**Results:**

The removal torque values did not reveal significant statistical differences between SAE-HD and SAE implants at any observation times (*P* = 0.06). Although a slight increase over time could be observed in both test and control groups. SAE-HD showed higher removal energy at 4 weeks (999.35 ± 924.94 × 10^− 2^ J) compared to that at 2 weeks (421.94 ± 450.58 × 10^− 2^ J), while SAE displayed lower values at the respective healing periods (*P* = 0.16). Regarding connection stiffness, there were no significant statistical differences neither within the groups nor over time. There was a strong, positive monotonic correlation between removal torque and removal energy (=0.722, *n* = 19, *P* < 0.001).

**Conclusions:**

In this study, no significant differences were observed between the specific hydrophilic (SAE-HD) and hydrophobic (SAE) surfaces evaluated, in terms of biomechanical properties during the early osseointegration period.

## Background

The progressive evolution of oral implant surface technology (i.e., micro to nanotopography and chemical composition) [1, 2], implant macrogeometry, surgical procedures [3–5], and loading protocols [6–8] has resulted in high survival and clinical success rates [9]. Accordingly, chemically active micro and nanostructured implant surfaces, presenting moderate surface roughness (*R*_a_/*S*_a_ values between 1 and 2 μm), enhance host-to-implant interactions [10–12] and have been shown to shorten the period needed for time-critical functional implant loading (i.e., so-called immediate or early loading) [13–15].

Recent in vitro analyses support the concept that hydrophilic surfaces upregulate the expression of angiogenic factors, activate the production of anti-inflammatory factors, and downregulate the expression of pro-inflammatory cytokines by osteoblasts [16] and macrophage-like cells [17, 18], and regulate osteogenic differentiation and maturation of mesenchymal stem cells (MSCs) [10, 19, 20] and human osteoblast-like cells [21–23], increasing osteogenesis and decreasing osteoclastogenesis [11, 23, 24]. Furthermore, higher surface energy and hydrophilicity is demonstrated to induce faster bone-to-implant contact (%BIC) and bone density, both in preclinical in vivo experiments [25–27] and in patients [7, 28, 29]. Indeed, higher biomechanical stability as expression of primary and secondary bone anchorage is recorded following hydrophilic implant placement [26, 30].

Particularly, a superhydrophilic moderately rough titanium (Ti) implant surface (contact angle less than 5°) has been suggested to play a critical role during the early healing period [27] and establishment of a successful osseointegration [31] in preclinical in vivo studies. The microarchitecture and density of the trabecular bone formed around oral implants, characterized by a high ratio of metabolic activity and remodeling, is one of the main determinants of interfacial shear strength, mechanical resistance, and adaptation to overloading stress [32]. The potential of chemically modified superhydrophilic implant surfaces to enhance osteogenic differentiation and increase early bone apposition onto the implant may shorten the implant stability drop, occurring due to bone remodeling during the first few weeks of implantation [13]. Consequently, such microstructured surface technology may lessen the healing (non-loading) period and allow more readily immediate or early functional implant loading in patients with reduced bone density. Nevertheless, there is lack of knowledge regarding the intrinsic biomechanical aspects of osseointegration related to this specific implant surface technology (i.e., removal torque, removal energy, and connection stiffness) during the initial osseointegration process.

Evaluation of biological effects and biomechanical properties of innovative technologies in the field of implant dentistry in preclinical animal models, prior to translational research, complies with standard regulations [33]. Thus, the aim of this preclinical animal study was to evaluate the effect of surface microtopography and chemistry on the biomechanical properties of implants with a sandblasted, dual acid-etched surface, with or without an additional chemical modification (SAE-HD^1^ and SAE,^2^ respectively), during the early stages of osseointegration in the tibia of Beagle dogs. Specifically, we hypothesized that a chemically active implant surface presenting superhydrophilicity generates higher interfacial shear strength in comparison to a hydrophobic surface, expressed as higher maximum removal torque, removal energy, and connection stiffness.

## Methods

The present preclinical in vivo study is reported according to the Animal Research: Reporting of In Vivo Experiments (ARRIVE) guidelines, in regard with relevant items [33]. The animal experimental protocol was approved by the Bioethics Committee for Animal Experimentation (CEUA, protocol no. 098/10) at the Federal University of Uberlândia and followed the normative guidelines of the National Council for Animal Control and Experimentation (CONCEA), constituent of the Ministry of Science, Technology and Innovation (MCTI), Law no. 11.794, 08/19/2008, Brazil. The in vivo part of the study was conducted between November 2012 and January 2013.

### Experimental units

Twenty-four commercially pure Ti implants (10 mm × 4 mm, *L* × Ø) with a moderately rough surface, produced by means of sandblasting and dual acid-etching with hydrochloric and sulfuric acid were used in the present study. Implants in the control group were only sandblasted and dual acid-etched (SAE; *n* = 12). In the test group, after sandblasting and dual acid etching, implants received proprietary technology treatment, including microwave treatment and insertion in isotonic saline solution resulting in significantly increased hydrophilicity (SAE-HD; *n* = 12). These specific implant surfaces exhibit the following 3D surface roughness parameters for SAE and SAE-HD, respectively: *S*_a_ 1.44 μm ± 1.15 and 1.26 μm ± 0.17; *S*_z_ 14.57 μm ± 1 and 16.20 μm ± 7.8; *S*_dr_ 1.51 and 1.21%; *S*_ds_ 658.67 1/mm^2^ ± 27.42 and 643.33 1/mm^2^ ± 37.74; and *S*_sk_ −0.51 and −0.43 [27]. The chemical composition of the different surfaces was examined by X-ray photoelectron spectroscopy (XPS). The atomic percentage values of carbon were 49 and 17, and 22 and 59 of oxygen in SAE and SAE-HD groups, respectively. The surface energy and wettability was investigated by means of a static contact angle analysis by means of the sessile drop technique [34] applying simulated body fluid (SBF) solution. SAE-HD disks reveal a superhydrophilic behavior (contact angle < 5°), whereas SAE surfaces display superhydrophobic properties (contact angle > 90°).

The implants present similar macrodesign with an external hexagon connection system and cylindrical body containing double triangular threads with high potential for bone compression recommended for type III and IV bone, commercially available as Titamax Ti Ex®. Neodent®^3^ supplied all implants.

### Animal model

Six male Beagle dogs (~ 1.5 years old), weighting between 13 and 15 kg, were used in the present study. All animals were acclimatized in the experimental animal care facility of Federal University of Uberlândia for 2 weeks previously to the experimental procedures and randomly pair-housed in standard shelters (1 × 1.5 m kennel) to allow environmental enrichment (i.e., variety of toys, daily group play sessions, resistance running, and training) at the ambient temperature of 22 °C, under controlled humidity and 12-h circadian rhythm. The diet consisted of hard pellet and water ad libitum. The animal caretakers were blind to the experimental groups. The level of pain, distress, or suffering was daily assessed during the observation period to ensure the welfare of the animals. Aiming to guarantee selection blindness during the experimental allocation, the groups were systematically coded by a third person and the first implant to be placed in each tibia was randomly assigned. Sample size was calculated based on information in previous studies using the dog tibia model to allow evaluation of possible differences in bone-to-implant contact between groups. The surgical procedures and the histological/histomorphometrical outcomes of the study have been reported elsewhere [31].

**Fig. 1:**
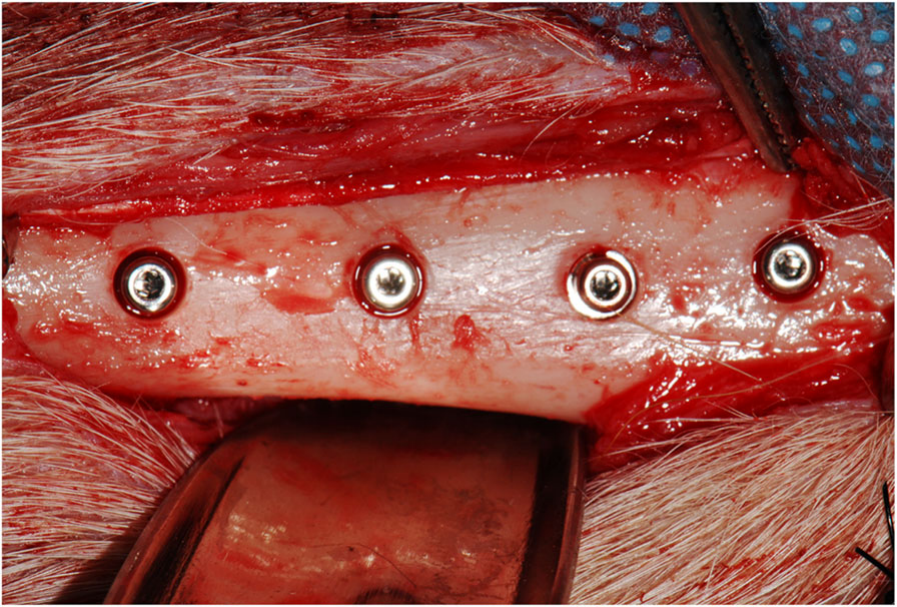
Two pairs of implants (10 mm × 4 mm, *L* × Ø) from each of the experimental groups were placed in each tibia with an alternating fashion in terms of medio-distal positioning regarding the group, but with the first group chosen at random. Implants were placed with an inter-implant distance of 1 cm

Briefly, two pairs of implants (10 mm × 4 mm, *L* × Ø) from each of the experimental groups were placed under copious sterile saline irrigation and with a torque of about 45 N cm (last drill 3.5 mm Ø) in each proximal tibia of the animals (total no. of implants 48). Implants were placed with an alternating fashion in terms of medio-distal positioning with the first group chosen at random and surgeries were staged between left and right tibia to provide 2 and 4 weeks of healing times, i.e., 12 implants per group and per observation time. The first implant was inserted ca. 2 cm below the femoral-tibial-patellar joint line at the central medial-lateral portion of the proximal tibiae (Fig. 1). The following implants were placed in a distal direction with inter-implant distances of 1 cm along the central region of the bone until the tibia patellar joint. Implants were furnished with cover screws and then soft tissues were sutured in layers for primary intention healing. Postoperative pain and infection control was provided for 7 days. The animals were euthanized under sedation with an anesthesia overdose, and the upper third of the tibias was retrieved. The specimens were fixed in 10% buffered formalin solution and half of them were allocated to histomorphometric analysis and the other half to biomechanical analysis, reported herein.

### Assessment of interfacial biomechanical properties

To assess the biomechanical strength of the bone-implant interface, the following parameters were assessed: (a) maximum removal torque (N cm) (primary outcome measure), obtained during the unscrewing process (primary outcome measure); (b) connection stiffness (N cm/rad), corresponding to the ratio between removal torque and angular displacement (secondary outcome measure); and (c) removal energy (× 10–2 J), corresponding to the energy (workload) necessary to completely unscrew the implant (secondary outcome measure).

**Fig. 2:**
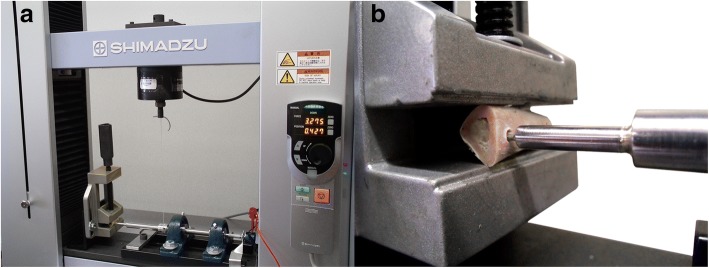
Adaptation of Shimadzu universal testing machine for performing removal torque test of dental implants. **a** General view. **b** Assembly detail of connection between Allen keys socket and the implant placed in the tibia

**Fig. 3:**
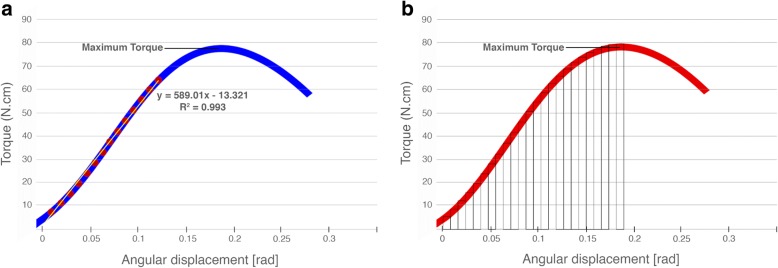
Representative curve of the torque test for implants. **a** Graph of torque versus angular displacement with linear regression curve, and equation, representing the connection stiffness. **b** Determination procedure of unscrewing implant work up to test’s maximum torque

**Fig. 4:**
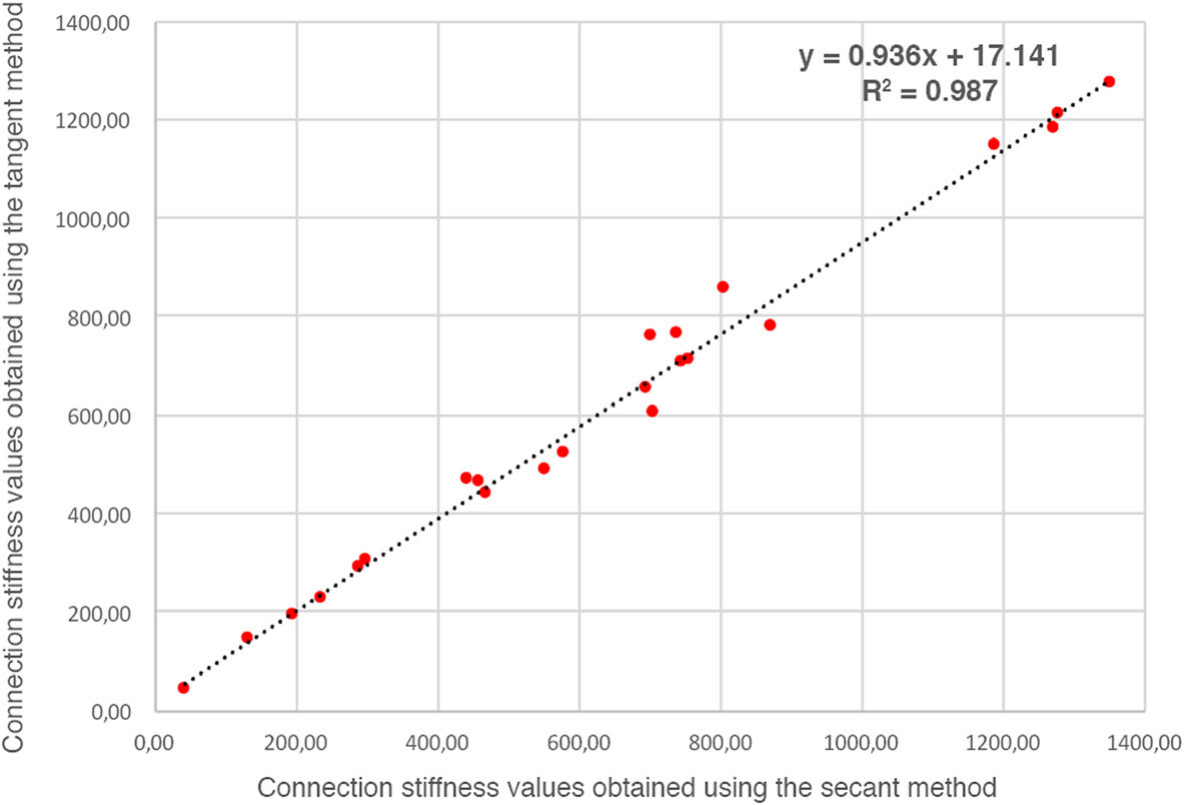
Comparison among secant and tangent methods to calculate the connection stiffness values, which reveals the absence of mathematical discrepancy

The removal torque test was conducted on a Shimadzu universal testing machine.^4^ This equipment was adapted in order to determine the referred properties (Fig. 2a, b). A horizontal shaft, supported by two ball bearings, with Allen keys socket on one end and a rotation sensor on the other end, was connected with a steel string to the mobile span of the Shimadzu universal testing machine, in such a way that the linear motion was converted to a rotational motion. The dog’s tibia bone block containing the implant was placed in alignment and inside the Allen keys socket and fixed with an adjustable clamp, in order not to rotate during the test. The upper span speed, at which the string was attached, was adjusted to produce a shaft rotation speed of 0.005 rad/s. During the test, both torque (N cm) and angular displacement (rad) were acquired using a sampling rate of 10 samples/s (Fig. 3a, b). In order to calculate the connection stiffness (N cm/rad), the tangent method was applied to the data after obtaining the linear correlation coefficient (*R*^2^) compared to the secant method, revealing the absence of mathematical discrepancy between the application of both methods (Fig. 4).

Calibration of one blinded examiner (R.N.R.J) and repeated measurements for data reproducibility was performed under supervision and prior to performing the removal torque test and respective calculation of connection stiffness.

### Statistical analysis

A general linear statistical model with torque, energy, and stiffness as dependent variables and implant surface and time in vivo as independent variables was used at 95% level of significance and performed by one-way ANOVA followed by Tukey’s post hoc test. The Spearman rank correlation test was taken in order to test the association concerning the investigated dependent variables. Since sample size calculation, as already mentioned, was made to allow evaluation of possible differences in bone-to-implant contact between groups, a post hoc analysis was performed to define the minimum detectable difference between groups regarding the parameters assessed herein, with a power of 80% and an alpha error of 0.05%. The IBM® SPSS® Statistics software^5^ was used.

## Results

**Fig. 5:**
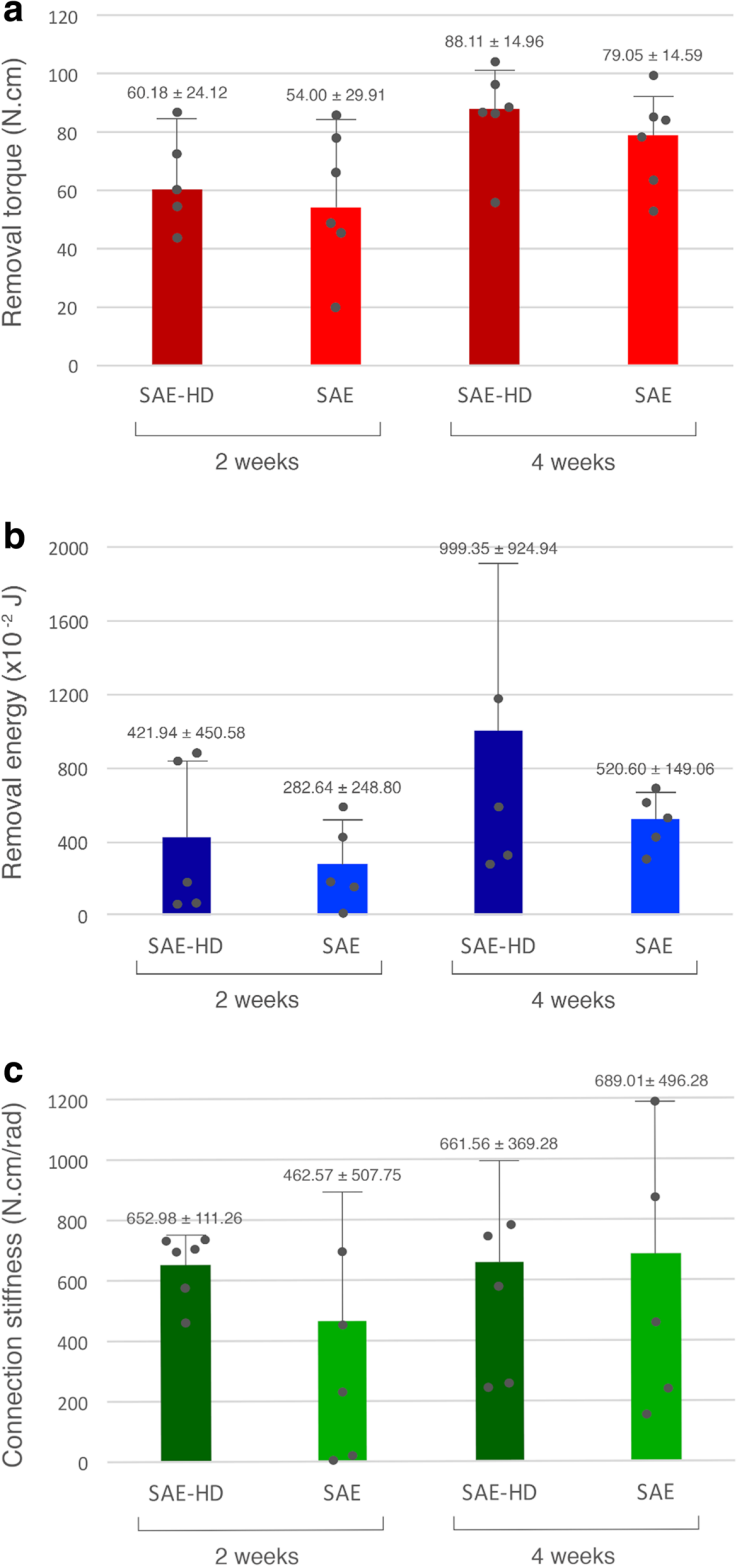
Mean and standard deviation of the biomechanical data at both observation periods (*P* > 0.05). **a** Removal torque. **b** Removal energy. **c** Connection stiffness

No remarkable events were observed during the surgical procedures and the subsequent healing period. The relative biomechanical performance of both experimental implant surfaces is illustrated in a representative graph of removal torque versus angular displacement. The removal torque, removal energy, and connection stiffness values are described in terms of mean, standard deviation, and 95% confidence interval for mean in Fig. 5. The results of one-way ANOVA variance and Tukey’s post hoc test values of the variables for SAE-HD and SAE implant at 2 and 4 weeks postoperatively (*n* = 6) are demonstrated in Table 1.

**Table 1:** One-way ANOVA variance and Tukey’s post hoc test values of removal torque (N cm), removal energy [N cm/rad (0.01 J)], and connection stiffness [N cm/rad] for SAE-HD and SAE implants at 2 and 4 weeks postoperatively (*n* = 6; *P* < 0.05)

		Sum of squares	df	Mean square	*F*	Sig. (*P* value)
Removal torque	Between groups	4572.571	3	1524.190	3.186	0.06
	Within groups	9569.095	21	478.455		
	Total	14,141.666	24			
Removal energy	Between groups	1,245,769.635	3	415,256.545	1.677	0.16
	Within groups	3,715,217.914	16	247,681.194		
	Total	4,960,987.549	19			
Connection stiffness	Between groups	193,886.351	3	64,628.784	0.396	0.76
	Within groups	3,264,332.301	18	163,216.615		
	Total	3,458,218.652	21			

The removal torque values (N cm) did not reveal significant statistical differences between SAE-HD and SAE implants at any observation period (*P* = 0.06), but a slight increase over time could be observed in both test and control groups (Fig. 5a). Removal torque at 2 weeks was 60.18 ± 24.12 and 54.00 ± 29.91 N cm, and at 4 weeks was 88.11 ± 14.96 and 79.05 ± 14.59 N cm for SAE-HD and SAE implants, respectively (Fig. 5b). SAE-HD showed higher removal energy [J] at 4 weeks (9.99 ± 9.25) compared to 2 weeks (4.22 ± 4.50), while SAE displayed lower values at corresponding healing times (5.21 ± 1.49 vs. 2.83 ± 2.49, respectively) (*P* = 0.16). Regarding connection stiffness (N cm/rad), SAE showed lower values at 2 weeks (462.57 ± 507.75) compared with 4 weeks of healing (689.01 ± 496.28), however not statistically significant (Fig. 5c). SAE-HD disclosed similar results at 2 and 4 weeks (652.98 ± 111.26 vs. 661.56 ± 369.28, respectively) without statistically significant differences neither between the groups, nor over time (*P* = 0.76).

A Spearman’s correlation test was run to determine the connection between maximum removal torque, removal energy, and connection stiffness values (Table 2) during the interfacial strength assessment. There was a strong, positive monotonic correlation between removal torque and removal energy (=0.722, *n* = 19, *P* < 0.001). However, there was no evidence of positive correlation between removal torque and connection stiffness (=0.352, *n* = 24, *P* = 0.092). Moreover, a negative correlation between removal energy and connection stiffness was observed (=− 0.91, *n* = 19, *P* = 0.710).

**Table 2:** Spearman rank correlation coefficient values between removal torque (N cm), removal energy [N cm/rad (0.01 J)], and connection stiffness [N cm/rad] for SAE-HD and SAE implants at 2 and 4 weeks postoperatively (*n* = 6; *P* < 0.01)

			Removal torque	Removal energy	Connection stiffness
Spearman’s rho	Removal torque	Coefficient	1.000	0.722	0.352
		Sig. (two-tailed)		0.000	0.092
		*N*	24	24	24
	Removal energy	Coefficient	0.722	1.000	(−) 0.091
		Sig. (two-tailed)	0.000		0.710
		*N*	19	19	19
	Connection stiffness	Coefficient	0.352	(−) 0.091	1.000
		Sig. (two-tailed)	0.092	0.710	
		*N*	21	21	21

## Discussion

Improving surface wettability aims to increase the implant surface area achieving most favorable protein adsorption and cellular adhesion and thereby to positively regulate the biological response at the initial osseointegration process. Thus, the superior potential of superhydrophilic surfaces in enhancing osseointegration at early stages of bone formation may also enhance their load-bearing capacity and biomechanical resistance.

In the present study, both SAE-HD implants and SAE implants showed relatively high amounts of maximum removal torque values at both observation times. In contrast, the SAE-HD implants showed relatively high values in removal energy compared with SAE implants at both 2 and 4 weeks. Specifically, the test group presented consistently higher values (about 100% higher) in removal energy compared with the control group at both observation times. Further, SAE-HD implants showed high values in connection stiffness already after 2 weeks of healing, while SAE implants required 4 weeks of healing to reach a similar level. Thus, despite the fact that the differences between the two groups were not significant for any of the evaluated parameters or observation times, the results seem to indicate that differences in surface properties between SAE-HD and SAE implants, somehow influenced osseointegration and intrinsic properties of shear strength. Indeed, greater removal torque values and interfacial stiffness for hydrophilic implants (modSLA)^1^ between 2 and 4 weeks, in comparison with SLA^2^, have been previously reported [35]. In this study [35], performed in the anterior maxilla of miniature pigs, hydrophilic implants revealed, on average, 8–21 and 9–14% significantly higher removal torque and interfacial stiffness values, respectively, than those of the SLA implants. Due to the remodeling process, the biomechanical parameters decreased with time for both implant surfaces, reflecting the developing biological stability.

It has been previously reported the existence of a correlation between removal torque and %BIC values [26], although the nature of these parameters differs from one another (three-dimensional versus most often two-dimensional parameter) [36]. Indeed, the lack of differences between the groups herein reflect well the results of the histomorphometric analysis of the other half of implants in the present study, reported elsewhere [31]. In particular, similar amounts of osseointegration in terms of %BIC and bone density were observed in both groups (SAE-HD vs. SAE) at each observation time, and there were no statistically significant differences regarding the respective parameters between the two observations times [31]. In contrast, Sartoretto et al. [27] demonstrated that Acqua® implants (Neodent®), which present similar technology as the SAE-HD implants, resulted in accelerated osseointegration when placed in tibia of rabbits after 2 weeks of healing, compared with implants with the Neoporos® surface, which in turn presents similar technology with the SAE surface. The difference between the study of de Jesus et al. [31] and Sartoretto et al. [27] in terms of the impact of surface technology on histomorphometric osseointegration parameters may be due to anatomical and/or biological differences in the experimental models employed. In this context, although there is no scientific support regarding the optimal experimental model to evaluate aspects of osseointegration, the dog is one of the most commonly used animal platforms [36]. The mandible of dogs is the most frequent location; however, a high percentage of studies on implant integration have used extra-oral implant sites, including the tibia [37]. It is suggested that due to its anatomy, with a large lumen and relatively low trabecular density [38], it possesses high discriminating potential regarding the impact of implant surface technologies to enhance osseointegration. Furthermore, the tibia allows placement of a larger number of implants comparing with the mandible, thus allowing the use of fewer animals and/or multiple types of comparisons/tests (e.g., biomechanical and histological evaluation).

On the other hand, lack of significant differences between the two groups in the present experiment could be due to the fact that the effect of hydrophilicity, in terms of accelerating bone healing and osseointegration, was unfolded before the first evaluation time-point of 2 weeks, i.e., during the very early healing period. Consistently, pre-clinical investigations show the potential of chemically modified surfaces to rapidly modulate the host-to-implant response upon prompt adsorption of blood proteins [20, 22]. In a recent study reported by Vasak et al. [39], hydrophilic implants (SLActive^a^) strengthened the apposition of newly formed bone at the very early healing period between 5 and 10 days in the intra-oral model of minipigs, even though they did not reveal significant statistical differences in comparison to hydrophobic surfaces (SLA). Clinically, a systematic review of human histological studies on molecular aspects of osseointegration [29] has shown that moderately rough implant surfaces with high hydrophilicity enhance molecular processes related to osseointegration during the early stages of wound healing. Similar clinical studies assessed the degree of new bone-to-implant contact (%NBIC) around SLActive implants in comparison to SLA placed in the mandibular retromolar region in man during the early stages of osseointegration [28, 40]. The authors reported a progressive increase in %NBIC around both implants, whereas chemically active surfaces disclosed higher values at 2 and 4 weeks compared with SLA, no longer observed after 6 weeks of osseointegration.

Studies including biomechanical analysis of osseointegration are usually assessing maximum removal torque. Although this parameter is important, use of a single parameter appears not sufficient for a complete biomechanical assessment of osseointegration. Herein, connection stiffness, reflecting the rigidity (i.e., the stability of the implant under load), and removal energy, reflecting the overall energy (workload) necessary to loosen the bone-to-implant connection, were additionally assessed. It is suggested that the use of these three parameters in a complementary manner is essential for complete evaluation of the biomechanical properties of implants. Indeed, two different osseointegrated implants can have the same maximum removal torque, but distinct connection stiffness; similarly, they may show the same removal energy but perform in a very distinct fashion and show distinct connection stiffness and maximum removal torque. To the best of the authors’ knowledge, this is the first original research reporting removal energy as an intrinsic removal torque property in relation to connection stiffness during biomechanical assessment of hydrophilic implants; however, removal energy has already been used assessing mini-implants with hydrophobic surfaces [41].

In fact, the observed lack of differences between the groups could be attributed to the relatively low number of specimens per group. According to the observed data herein, applying a high power (80%) with the present sample size would had revealed a relatively large difference in removal torque equivalent to 43% between the experimental groups. Regarding removal energy and connection stiffness, this difference would had been equal to 71 and 34%, respectively. In addition, a significant difference may not had been achieved due to the nature of the specimens. Biological material properties vary greatly from organism to organism and even in the same animal. Therefore, in future studies when using the presented methodology, a greater number of specimens must be considered. This will evidence a superior discrimination capacity. Nonetheless, the current methodology is far more reliable than the use of a single parameter methodology.

## Conclusions

No significant differences were observed between the specific hydrophilic (SAE-HD) and hydrophobic (SAE) surfaces evaluated in this study, in terms of biomechanical properties during the early osseointegration period.

## Footnotes

1 Chemically modified sandblasted, large grit, and acid-etched surfaces

2 Sandblasted, large grit, and acid-etched surfaces

3 Neodent, Rua Benjamin Lins, 742, Curitiba, PR 80420–100, Brazil

4 Shimadzu Corporation, Nishinokyo Kuwabara-cho, Nakagyo-ku, Kyoto 604–8511, Japan

5 IBM Corporation v.23, 1 New Orchard Road, Armonk, New York 10, 504–1722, USA
